# Defining a core outcome set for hypermobility spectrum disorders and hypermobile Ehlers-Danlos syndrome: A Delphi consensus study

**DOI:** 10.1007/s10067-024-07172-3

**Published:** 2024-10-09

**Authors:** Natalie L. Clark, Melissa Johnson, Amar Rangan, Lucksy Kottam, Andrea Hogarth, Sarah Scott, Katherine Swainston

**Affiliations:** 1 South Tees Hospitals NHS Foundation Trust, Middlesbrough, UK; 2 Department of Health Sciences, University of York, York, UK; 3 School of Psychology, Faculty of Medical Sciences, Newcastle University, Newcastle Upon Tyne, UK

**Keywords:** Core outcome set, Ehlers-Danlos syndrome, Hypermobility, Measurement, Survey

## Abstract

The reported prevalence of hypermobility spectrum disorders (HSD) and hypermobile Ehlers-Danlos Syndrome (hEDS) is unclear due to complex presentations and lack of awareness amongst professionals, contributing to diagnostic and management uncertainties. Standardising an outcome measure to assess symptoms most important to patients should help determine the impact of interventions. This Delphi study aims to reach a consensus with stakeholders on the core outcome set for children and adults with HSD/hEDS. A three-round modified Delphi consensus study with a follow-up consensus meeting was used. Stakeholder groups consisted of (1) individuals with HSD/hEDS; (2) family/friends/carers and (3) healthcare professionals. Participants could belong to more than one stakeholder group. They rated 74 symptoms using a 9-point Likert scale: 1 “not important to 9 “critically important”. Symptoms achieving a consensus rating of ≥ 70.0% critical importance across all groups were included. In Round 1, 766 responses were received from 600 participants, reducing to 566 responses from 438 participants by Round 3, with 53 participating in the consensus meeting. Overall, 30 symptoms met the ≥ 70.0% critically important threshold to be included in the final core outcome set. These were categorised under the specialties of musculoskeletal and orthopaedics, social, pain, gynaecology and urology, negative affect, neurological, gastrointestinal and “other”. This study is the first to identify by consensus the core outcome set to be measured for patients with HSD/hEDS. The importance of these outcomes was confirmed by individuals living with the condition, their family, friends, carers and relevant healthcare professionals. Trial registration: IRAS ID: 326,855; East Midlands — Leicester South REC (reference: 23/EM/0143); Protocol registered with the COMET Initiative.

**Table:** 

Key Points *• There is a lack of standardised outcome measure for HSD/hEDS research studies due to the heterogeneity of symptom presentations.* *• Symptoms across musculoskeletal and orthopaedics, social, pain, gynaecology and urology, negative affect, neurological, gastrointestinal and “other” specialties were identified as the core outcome set defined as* ≥ *70.0% critically important to measure for individuals with HSD/hEDS.* *• There were a significant number of symptoms, widely recognised in the literature to be comorbid to HSD/hEDS, that reached a critical importance threshold of 50.0–69.9%, restricting the core outcome set to only those that met 70% or above may be limiting.*

**Supplementary Information:**

The online version contains supplementary material available at 10.1007/s10067-024-07172-3.

## Introduction

Hypermobility spectrum disorders (HSD) and hypermobile Ehlers-Danlos syndrome (hEDS) are inherited connective tissue disorders primarily characterised by joint hypermobility and skin hyperextensibility [1, 2]. The reported prevalence of the conditions is unclear within the literature, though it is expected to be higher than reported due to difficulties and inaccuracies with diagnosis [3]. It is not uncommon for individuals to wait almost 20 years to be diagnosed with either one of the two conditions due to the variability in presentations and complex nature of symptoms [4]. Over recent years, there has been a growing recognition and research into the multisystemic symptoms and comorbidities of HSD/hEDS [5].

Most recently, a scoping review identified all the known physical, psychological and social implications of HSD/hEDS within an adult population using the biopsychosocial model, as reported in the literature over the last decade [6]. The review identified over 400 symptoms and conditions associated with HSD/hEDS in adults. Examples of common symptoms included musculoskeletal (MSK) pain [3, 7, 8], abdominal pain and nausea [9], with depression and anxiety also more prevalent [3, 8, 10, 11] as well as disruptions to education and employment [12]. Alongside literature reviews, a wealth of qualitative research has comprehensively documented patient perspectives and experiences of healthcare services and interactions [13, 14]. Interviews highlight the predominantly negative interactions across a range of healthcare professionals and describe instances of being mislabelled as a hypochondriac, misdiagnosed as having a mental health condition or growing pains [13, 15]. Attitudes of healthcare professionals are perceived as dismissive and lacking in compassion and understanding [15]; these attitudes could be attributable to poor knowledge of the conditions. However, once a diagnosis is obtained, individuals reported feeling psychologically validated, removing the uncertainties surrounding their symptoms. It also enabled them to receive the appropriate management for their condition, associated symptoms and comorbidities [16].

Currently, HSD/hEDS research studies lack a standardised outcome measure due to the heterogeneity of symptom presentations. Determining the impact of interventions on symptoms and appropriate pathways is therefore difficult in the absence of a robust measure, further impacting on the ability to influence policy and practice [17]. A Delphi consensus survey previously conducted in France aimed to determine the symptoms which had the greatest impact on patients diagnosed with hEDS or other Ehlers-Danlos syndrome subtypes [18]. In the first round, 118 patients responded, reducing to 87 by the final round. The survey concluded that pain, fatigue, sleep and MSK disorders had the greatest impact, despite a lack of consensus. Palmer et al. [19] developed a questionnaire to assess the impact of joint hypermobility syndrome (JHS) via a three-stage process with JHS patients. Though it demonstrated good concurrent validity, it was developed with patients alone. Collaborative working (e.g. with professionals) can have benefits such as improved patient care and outcomes, increased perspective and trusting patient-professional relationships [20]. Physiotherapists have since tested the measure, responding positively to the development of this toolkit [21]; however, this measure has a focus on physical functioning, lacking acknowledgement of the psychological attributes. Furthermore, the toolkit needs to be assessed as to whether it is applicable to the new terminologies HSD and hEDS [21]. “The Spider” is a new screening tool currently being developed with individuals with HSD/hEDS to assess the impact of symptomatic hypermobility and whether presenting this impact using a visual method has additional benefits [22].

To address the heterogeneity of symptoms and their reporting in previous studies, a core outcome set (COS) needs to be defined (Stage 1), followed by identifying the most appropriate measurement instruments for the COS (Stage 2) [23]. This paper reports the results of Stage 1, whereby the aim was to reach consensus on the most important outcomes for children and adults with HSD/hEDS, agreed by key stakeholders including patients with HSD/hEDS, their family, friends and carers and healthcare professionals.

## Materials and methods

### Design

A three-round modified Delphi consensus process was used, followed by a consensus meeting, as recommended by the Core Outcome Measures in Effectiveness Trials (COMET) Handbook [24]. The 404 symptoms and conditions identified from the scoping review [6] were used to inform the COS items. Duplicate symptoms as well as conditions that already had validated measures were removed, and related symptoms were grouped to avoid an exhaustive list that would cause participant fatigue when completing the survey.

A 9-point Likert scale, recommended by the Grading of Recommendations, Assessment, Development and Evaluations (GRADE) working group [25, 26], was used by participants to rate the importance of these symptoms to HSD/hEDS. This was graded as 1 to 3 = “not important”, 4 to 6 = “important but not critical” and 7 to 9 = “critically important”. Not applicable or not sure was also an option for instances whereby male individuals with HSD/hEDS are unable to grade importance of gynaecological symptoms, for example. The online survey was designed using the Research Electronic Data Capture (REDCap) platform, hosted by South Tees Hospitals NHS Foundation Trust.

### Setting and participants

Participants consisted of three stakeholder groups, predominantly residing in the United Kingdom (UK), i.e. (1) individuals over the age of 16 years with a clinical diagnosis of HSD/hEDS; (2) family members, friends or carers of individuals with HSD/hEDS and (3) healthcare professionals working with or have experience with HSD/hEDS patients. The survey design allowed participants to select more than one option (i.e. a participant could be an individual with HSD/hEDS and a professional who works with HSD/hEDS).

We anticipated a minimum recruitment of 40 participants overall with the majority expected to be individuals with HSD/hEDS. Though a larger sample size was welcomed to ensure adequate representation of participants from each key stakeholder group, participants were identified and invited via email, professional societies, research user groups and online platform advertisements.

### Procedure

**Fig. 1:**
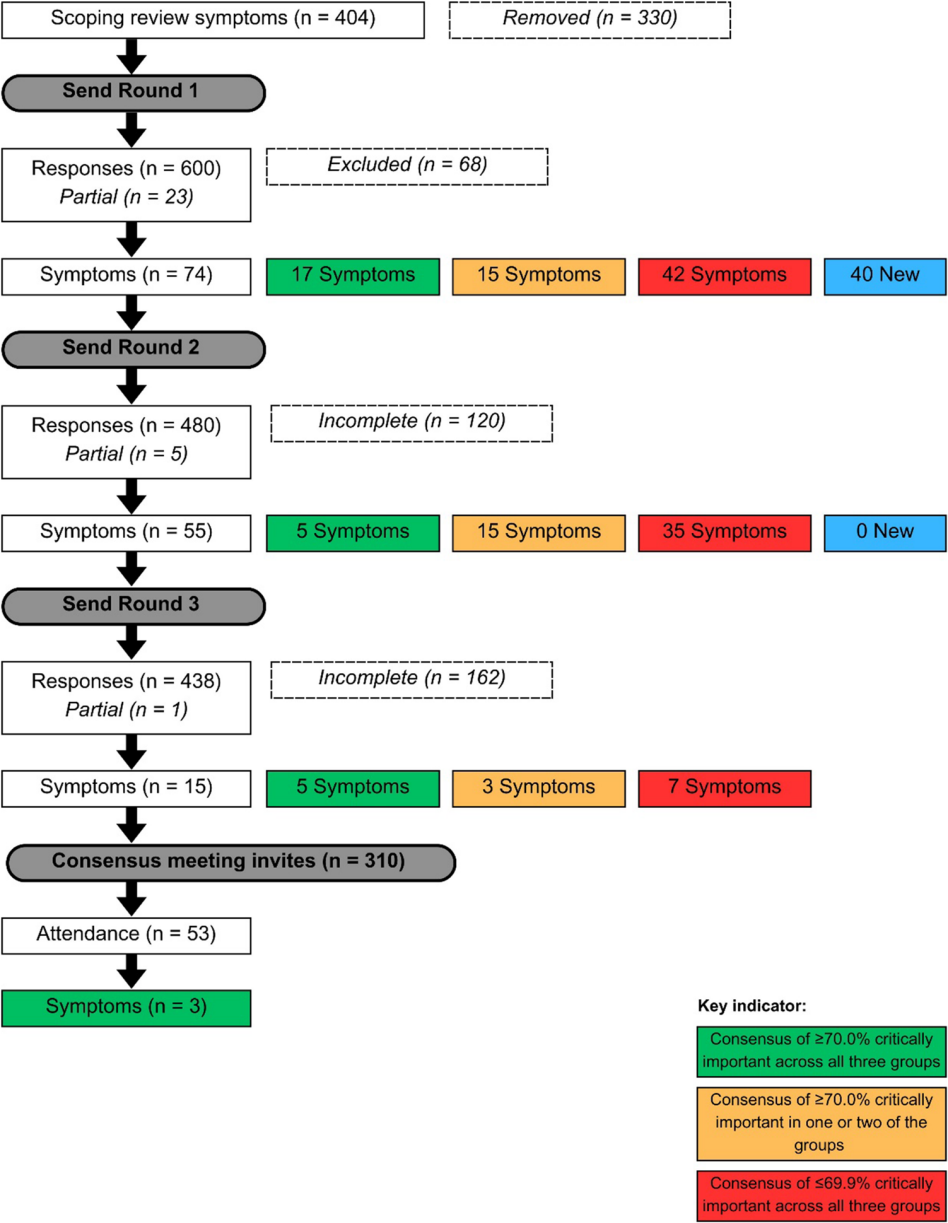
Flowchart of the Delphi consensus procedure

The REDCap survey link containing the participant information sheet (PIS), online consent form and Round 1 of Stage 1 was disseminated via email and social media platforms from 24 July 2023 to 31 August 2023. The Round 2 REDCap link was sent to participants between 27 September 2023 and 30 October 2023, followed by Round 3 between 13 November 2023 and 11 December 2023. The follow-up consensus meeting was held on 25 January 2024 with participants who indicated they would like to participate and had completed all three rounds. See Fig. 1 of the Delphi consensus process by round.

All rounds were open for a minimum of 4 weeks, with each survey taking no longer than 30 min to complete. Weekly reminders were sent to follow up partial responses and non-responders. Participants were provided a copy of their individual responses following the end of each round and were also sent the group responses from each round.

Participants who completed all three rounds were entered into a prize draw to win one of five £100 gift vouchers. Healthcare professionals were offered contributor acknowledgement upon completion of three rounds, with their consent.

### Statistical analysis

Participant characteristics are descriptively reported, including mean, standard deviation and percentages as appropriate.

The consensus results of each symptom per round are reported per stakeholder group and overall. Symptoms were analysed for a consensus threshold of ≥ 70% to be rated as “critically important”. The following rules were applied to the consensus analysis:

- Consensus of ≥ 70.0% critically important across all three groups was included in the final COS.
- Consensus of ≥ 70.0% critically important in one or two of the groups was re-rated in a subsequent round.
- Consensus of ≤ 69.9% critically important across all three groups was removed with progressive rounds of Delphi.

### Patient and public involvement

Two patient co-applicants from South Tees Hospitals NHS Foundation Trust’s established national hypermobility research user group were involved in the design and development of the research study and survey.

### Ethics and informed consent

Ethical approval was obtained from East Midlands — Leicester South Research Ethics Committee (REC) [REC reference: 23/EM/0143]. The protocol was registered as part of the COMET initiative prior to distributing the survey [23]. All participants were required to read a PIS and provide electronic written informed consent.

## Results

### Participant characteristics

Overall, 668 participants responded to the online survey; 68 were excluded due to an incomplete consent form or only completing the demographic section of the Round 1 survey. This left 600 participants, totalling 766 responses across the three groups, i.e. 506 individuals with HSD/hEDS (66.1%), 140 family, friends or carers (18.3%) and 120 professionals (15.7%). Of the 600 participants, 143 belonged to either 2 or 3 of the stakeholder groups.

The average age of participants was 40 years (range 18 to 77 years), with the majority being female (94.7%), White (95.7%) and from the UK (94.5%). Of the individuals with HSD/hEDS, 47.4% had HSD and 47.0% had hEDS. See Table 1 for participant characteristics.

**Table 1:** Participant characteristics

			Total		Individuals with HSD/EDS			Family, friends or carers			Professionals	
			(*n* = 600)		(*n* = 506)			(*n* = 140)			(*n* = 120)	
Age	Mean	(*SD*)	40	(12)	39	(12)	42		(12)	40		(12)
	Range		16 to 77	-	16 to 77	-	16 to 74		-	23 to 69		-
Sex	Female	(%)	568	(94.7)	487	(96.2)	134		(95.7)	109		(90.8)
Race/ethnicity	White	(%)	574	(95.7)	488	(96.4)	135		(96.4)	112		(93.3)
	Asian/Asian British	(%)	12	(2.0)	6	(1.2)	1		(0.7)	7		(5.8)
	Mixed	(%)	9	(1.5)	8	(1.6)	2		(1.4)	1		(0.8)
	Other	(%)	2	(0.3)	1	(0.2)	1		(0.7)	-		-
	Prefer not to say	(%)	2	(0.3)	2	(0.4)	1		(0.7)	-		-
	Unknown	(%)	1	(0.2)	1	(0.2)	-		-	-		-
Country of residence	UK	(%)	567	(94.5)	480	(94.9)	129		(92.1)	111		(92.5)
	Australia	(%)	3	(0.5)	1	(0.2)	2		(1.4)	2		(1.7)
	Canada	(%)	1	(0.2)	-	-	-		-	1		(0.8)
	USA	(%)	6	(1.0)	6	(1.2)	2		(1.4)	1		(0.8)
	India	(%)	1	(0.2)	-	-	-		-	1		(0.8)
	Unknown	(%)	22	(3.7)	19	(3.8)	7		(5.0)	4		(3.3)
Hypermobility diagnosis*	HSD	(%)	240	(40.0)	240	(47.4)	41		(29.3)	36		(30.0)
	hEDS	(%)	238	(39.7)	238	(47.0)	40		(28.6)	28		(23.3)
	EDS or other subtype	(%)	15	(2.5)	15	(3.0)	5		(3.6)	4		(3.3)
	Other	(%)	13	(2.2)	13	(2.6)	4		(2.9)	1		(0.8)
Education level*	Secondary school	(%)	28	(4.7)	28	(5.6)	7		(5.0)	-		-
	Further education, college	(%)	121	(20.2)	121	(23.9)	21		(15.0)	3		(2.5)
	University — undergraduate	(%)	178	(29.7)	178	(35.2)	26		(18.6)	23		(19.2)
	University — postgraduate	(%)	157	(26.2)	157	(31.0)	33		(23.6)	39		(32.5)
	Other	(%)	16	(2.7)	16	(3.2)	3		(2.1)	4		(3.3)
	Prefer not to say	(%)	6	(1.0)	6	(1.2)	-		-	-		-
Employment status*	Full-time	(%)	255	(42.5)	255	(50.4)	34		(24.3)	45		(37.5)
	Part-time	(%)	110	(18.3)	110	(21.7)	27		(19.3)	13		(10.8)
	Carer	(%)	8	(1.3)	8	(1.6)	4		(2.9)	-		-
	Student	(%)	26	(4.3)	26	(5.1)	5		(3.6)	5		(4.2)
	Unemployed	(%)	16	(2.7)	16	(3.2)	1		(0.7)	-		-
	Retired/medically retired	(%)	18	(3.0)	18	(3.6)	5		(3.6)	2		(1.7)
	Unable to work	(%)	63	(10.5)	63	(12.5)	12		(8.6)	1		(0.8)
	Other	(%)	8	(1.3)	8	(1.6)	2		(1.4)	1		(0.8)
	Prefer not to say	(%)	2	(0.3)	2	(0.4)	-		-	-		-
Setting**	Primary care	(%)	38	(6.3)	21	(4.2)	8		(5.7)	38		(31.7)
	Secondary care	(%)	58	(9.7)	31	(6.1)	13		(9.3)	58		(48.3)
	Intermediate care	(%)	6	(1.0)	3	(0.6)	1		(0.7)	6		(5.0)
	Other	(%)	27	(4.5)	17	(3.4)	9		(6.4)	27		(22.5)
Length in role**	0–1 years	(%)	14	(2.3)	9	(1.8)	6		(4.3)	14		(11.7)
	1–5 years	(%)	46	(7.7)	27	(5.3)	11		(7.9)	46		(38.3)
	5–10 years	(%)	25	(4.2)	14	(2.8)	6		(4.3)	25		(20.8)
	10 + years	(%)	35	(5.8)	19	(3.8)	7		(5.0)	35		(29.2)
Hypermobility training**	Yes	(%)	66	(11.0)	27	(5.3)	12		(8.6)	66		(55.0)
	No	(%)	54	(9.0)	42	(8.3)	18		(12.9)	54		(45.0)

*hEDS* hypermobile Ehlers-Danlos syndrome, *HSD* hypermobility spectrum disorder

* Asked only of individuals with HSD/hEDS; ** asked only of professionals who work with HSD/hEDS

The mean age at first onset of symptoms amongst individuals with HSD/hEDS was 11 years (range 0 to 55 years), with mean age at diagnosis of 29 years (range 0 to 69 years). Comorbidities were self-reported by individuals with HSD/hEDS and reported by family, friends or carers of the individual with HSD/hEDS. Amongst the most common were postural orthostatic tachycardia syndrome (POTS), asthma, anxiety, fibromyalgia, depression, irritable bowel syndrome (IBS), autism spectrum disorder (ASD), migraine, attention deficit hyperactivity disorder (ADHD), osteoarthritis and mast cell activation syndrome (MCAS).

Of the family, friends or carer group, the individual they completed it on behalf of was predominantly a daughter (followed by son, sister and mother), with the youngest current age of the individual starting from 4 years old. Of the professional group, the roles were wide-ranging with physiotherapists mostly completing the survey, followed by nurse or healthcare assistant and occupational therapists, with only 55% of the professionals previously receiving training on hypermobility.

### Round 1

Of the 766 responses from 600 participants and 74 symptoms presented, 17 (23.0%) met the consensus threshold for being critically important; 15 (20.3%) needed to be re-rated in the next round, and 42 (56.8%) did not reach the consensus threshold across any group. There were 40 common symptoms recorded amongst the free-text responses to be added to the next round, leaving 55 to rate in Round 2.

### Round 2

Following the analysis of Round 2, there were 619 responses (80.8%) from 480 participants (80.0%), 409 individuals with HSD/hEDS (80.8%), 114 family, friends or carers (81.4%) and 96 professionals (80.0%), in comparison to Round 1.

Of the 55 symptoms, five (9.1%) met the consensus threshold for being critically important; 15 (27.3%) needed to be re-rated in the next round, and 35 (63.6%) did not reach the consensus threshold across any group. There were no common symptoms recorded in the free-text responses to be added to the next round, leaving 15 to re-rate in Round 3.

### Round 3

Following analysis of Round 3, there were 566 responses (73.9%) from 438 participants (73.0%), 376 individuals with HSD/hEDS (74.3%), 101 family, friends or carers (72.1%) and 89 professionals (74.2%), in comparison to Round 1.

Of the 15 symptoms, five (33.3%) met the consensus threshold for being critically important; three (20.0%) required further discussion in the consensus meeting; and seven (46.7%) did not reach the consensus threshold across any group.

### Follow-up consensus meeting

Participants who had consented to participating in the consensus meeting and completed three rounds were invited to take part. In total, 310 were invited, with 53 attending. These were mostly individuals with HSD/hEDS with smaller representations from the other two groups. Attendees were presented with the study findings to date with anonymous feedback documented. Those attending the meeting agreed that the three symptoms to be re-rated would be determined via majority vote overall, despite the professional group rating two of the symptoms lower.

### Final core outcome set

**Fig. 2:**
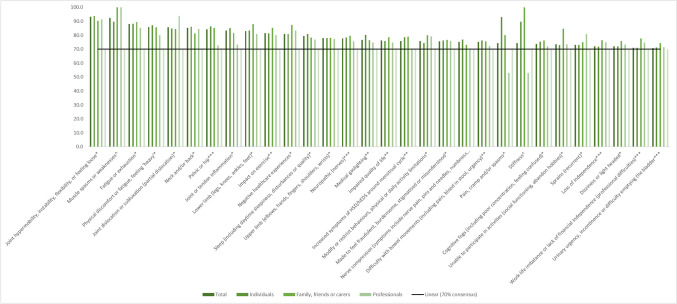
A bar chart representing the symptoms that met consensus at ≥ 70% critically important or above the linear treadline, in total and by group

The results of symptoms meeting the consensus threshold of ≥ 70.0% critical importance to be included in the COS are represented in Fig. 2.

This included 30 symptoms across eight specialties:

1. *MSK and orthopaedics (eight symptoms):* joint hypermobility, instability, flexibility or feeling loose; muscle spasms or weaknesses; physical discomfort or fatigue, feeling ‘heavy’; joint dislocation or subluxation (partial dislocation); joint or tendon inflammation; nerve compression (symptoms include nerve pain, pins and needles and numbness); stiffness; and sprains (recurrent).
2. *Social aspects (eight symptoms)*: impact on exercise; negative healthcare experiences; impaired quality of life; modified or restricted behaviours, physical or daily activity limitations; unable to participate in activities (social functioning and abandoning of hobbies); loss of independence; work-life imbalance or lack of financial independence (professional difficulties).
3. *Areas of pain (five symptoms)*: neck and/or back; pelvic or hip; lower limb (legs, knees, ankles and feet); upper limb (elbows, hands, fingers, shoulders and wrists) and neuropathic (nerves).
4. *Gynaecology and urology (three symptoms)*: urinary urgency, incontinence or difficulty emptying the bladder; pain, cramp and/or spasms; and increased symptoms of HSD/hEDS around menstrual cycle.
5. *Negative affect (two symptoms)*: medical gaslighting; made to feel fraudulent, burdensome, stigmatised or misunderstood.
6. *Neurological (two symptoms)*: dizziness or lightheaded; cognitive fogs (including poor concentration and feeling confused).
7. *Other (two symptoms)*: sleep (including daytime sleepiness, disturbances or quality); fatigue or exhaustion.
8. *Gastrointestinal (one symptom)*: difficulty with bowel movements (including pain, blood in stool and urgency).

See Supplementary Material 1 for those that meet a consensus of threshold of 50 to 69.9%, as well as those that fall beneath a 49.9% consensus rating.

## Discussion

This paper has reported the findings of a large Delphi consensus study that aimed to define the most important symptoms to include as a COS for children and adults with HSD/hEDS, with implications for research and practice. Overall, 30 symptoms achieved the predefined consensus for being critically important following three Delphi rounds and a consensus meeting. These outcomes were determined by a stakeholder group consisting of patients, family, friends, carers and healthcare professionals. The results will be used to guide the next stage of the project, whereby the appropriate instruments to measure the COS will be determined.

With the Delphi consensus process in general, there is a lack of strict criteria for determining consensus, though most studies use a threshold of ≥ 70.0%. In the current study, there were a significant number of symptoms that reached between a 50.0 and 69.9% consensus threshold of critical importance, of which are widely recognised by the literature as comorbid to the condition. The lower rating may reflect the diversity of symptoms and varying impact and consequence from person to person [16]; this was supported by the consensus meeting discussions. Therefore, to measure only the symptoms that met ≥ 70.0% consensus may be limiting. The COMET handbook stipulates that the COS represents the minimum that should be measured and are most important to stakeholders [24]. Considering the relatively large outcome set with symptoms from multiple organ systems, it may be more appropriate to develop an outcome measure using computer-adaptive testing that allows personalisation and consideration of the additional critically important symptoms as found by the Delphi.

The items that made it to the final COS were largely influenced by the individuals with HSD/hEDS group as this was the largest group of respondents. Interestingly, the family, friends or carers group rated symptoms as more critically important than the other two groups. Previous qualitative research has underpinned the role of parents in the diagnosis and management of their children with HSD/hEDS [27]. The research describes the journey of parents researching the conditions to become experts and advocates for their children in the absence of professional understanding or adequate support from healthcare services. Likewise, individuals with HSD/hEDS have reported doing their own research to become more self-aware of their condition, enabling them to self-manage their symptoms more effectively [15]. The different ratings between these two groups could be attributed to how each group perceives these symptoms. As the symptoms of the conditions commonly begin in childhood, family, friends or carers have been observers from the beginning and potentially recall duration, severity and impact on childhood greater than the individuals themselves [27]. In comparison, individuals may have scored symptoms as less critically important as they have accepted these symptoms as part of their condition and learnt self-management techniques, such as pacing, to cope with activities of daily living [13, 28]. Though individuals may acknowledge that these symptoms are associated with the condition, the importance of their impact on daily life might be perceived as lower in comparison to other symptoms, as well as differing from person to person [16]. Individuals with HSD/hEDS described this as the reason for the lower rating within the consensus meeting. For example, dermatological symptoms did not meet a critically important consensus rating of more than 35% in individuals, compared to the MSK and orthopaedic manifestations and areas of pain that exceeded the consensus threshold.

Of the final COS, professionals only rated three as more critically important compared to the other two groups, these were within the MSK and orthopaedic specialty (muscle spasms or weaknesses; joint dislocation or subluxation and recurrent sprains). Professionals are seemingly well-informed and knowledgeable of the MSK manifestations of the conditions [29–31], including those that met consensus. However, they are less knowledgeable of the additional multi-systemic features and comorbidities, as demonstrated by the lower ratings of critical importance in the Delphi, and as supported by previous research [14, 29, 30]. Interestingly, the professionals often rated the psychological and negative affect related symptoms higher in critical importance than the individuals with HSD/hEDS group; for example, “feeling depressed (not a diagnosis of depression)” (61.3% vs 50.2%) and “feeling distressed” (66.1% vs 50.0%), respectively. Patients understandably present with accompanying psychological manifestations because of the severity of the physical symptoms, combined with negative healthcare experiences. It is therefore common for professionals to assume the psychological manifestations to be the primary concern, leading to misdiagnoses, as opposed to being secondary to the physical symptoms [13, 31].

Notably, the psychosocial factors meeting consensus across the three groups were negative healthcare experiences (ranked 11th), medical gaslighting (ranked 15th) and being made to feel fraudulent, burdensome, stigmatised or misunderstood (ranked 19th). Delays to diagnosis of HSD/hEDS are well documented; some reporting as long as 19 years with multiple healthcare encounters to have their symptoms validated [4]. These patients are often dismissed due to the condition being contested within the medical field [32]. One patient with hEDS has previously reported interactions with doctors as being so terrible; she received a clinical diagnosis of post-traumatic stress disorder, accompanied by therapy to address the psychological damage inflicted [15]. Granted, this is an extreme example. This dismissive attitude can be perceived as medical gaslighting whereby the professional may or may not be consciously viewing the patients’ complaints as subjective and downplaying the severity, as well as attributing them to an underlying psychological condition [33]. It goes to further emphasise the importance of a trusting professional-patient relationship, particularly when the patient can be viewed as an expert in their own condition [34]. These findings need to be approached with caution and sensitivity as to not add further tension between patients and healthcare professionals and the services. It adds to the urgent need to provide professionals with appropriate education and HSD/hEDS specific training. This is supported by our data that only 55% of professionals in our survey have previously received condition-specific training and with a previous survey finding only 51% of physiotherapists received the specific training [30].

### Limitations

There may be some limitations with the sample and sampling strategy, including inclusion bias. We did not deliberately ensure an equal representation within each group, though we did anticipate a larger proportion of individuals with HSD/hEDS. Despite opening the survey internationally, many participants were from the UK and findings may therefore reflect UK practice and healthcare experiences. Applicability to international settings may need a cautious approach. The sampling strategy and lack of geographical spread could have impacted on the number of individuals with HSD/hEDS responding from ethnic minority backgrounds; evidence indicating that those of an Asian background have a higher prevalence of hypermobility [29]. Diagnosis of HSD/hEDS was also self-reported; however, we did specify the participants must have received a clinical diagnosis. Those that stated they had not yet received a diagnosis were classified under “Other” (2.2%).

## Conclusions

The first stage of this project using the modified Delphi consensus study has successfully outlined the COS that met a ≥ 70.0% consensus of critical importance to be included in an outcome measure. The second stage will use the COS to determine which measurement instruments and scales can accurately assess these. The outcome measure will then be tested for validity and used to assess impact of interventions, management and care pathways for individuals with HSD/hEDS.

## Supplementary Information

Below is the link to the electronic supplementary material.

Supplementary file1 (DOCX 194 KB)

## Data Availability

The datasets analysed during the current study are available from the corresponding author upon reasonable request.

## Abbreviations

ADHD: Attention deficit hyperactivity disorder
ASD: Autism spectrum disorder
COMET: Core outcome measures in effectiveness trials
COS: Core outcome set
GRADE: Grading of recommendations, assessment, development and evaluations
hEDS: Hypermobile Ehlers-Danlos syndrome
HSD: Hypermobility spectrum disorders
IBS: Irritable bowel syndrome
JHS: Joint hypermobility syndrome
MCAS: Mast cell activation syndrome
MSK: Musculoskeletal
PIS: Participant information sheet
POTS: Postural orthostatic tachycardia syndrome
REC: Research Ethics Committee
REDCap: Research electronic data capture
UK: United Kingdom
