# Supplementary material for: Defining a core outcome set for hypermobility spectrum disorders and hypermobile Ehlers-Danlos syndrome: A Delphi consensus study
Source: Clin Rheumatol. 2024 Oct 9;43(12):3951–61. doi: 10.1007/s10067-024-07172-3 (PMC11582216; doi:10.1007/s10067-024-07172-3)
Supplement: Supplementary file 1 — Supplementary file1 (DOCX 194 KB) [file 10067_2024_7172_MOESM1_ESM.docx]

**Figure 3:** A bar chart representing the symptoms that met consensus between 50% and 69.9% critically important linear treadline, and 49.9% or below, in total and by group.


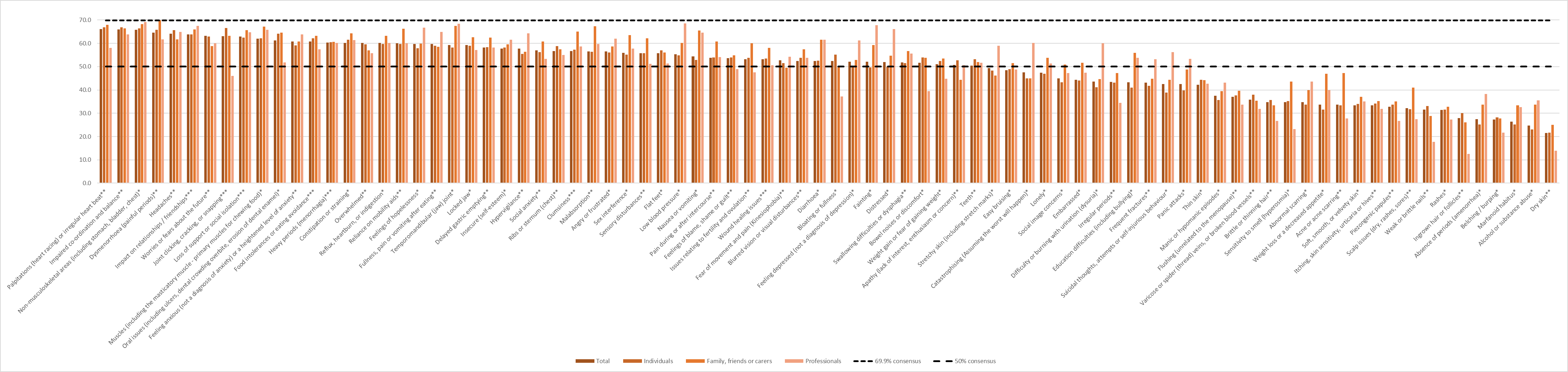


**Met consensus in Round 1; **Met consensus in Round 2; ***Met consensus in Round 3; ^Met consensus in Consensus Meeting*
